# *Mycobacterium sherrisii* Pulmonary Disease, Burkina Faso

**DOI:** 10.3201/eid2111.141809

**Published:** 2015-11

**Authors:** Emanuele Borroni, Gisèle Badoum, Daniela M. Cirillo, Alberto Matteelli, Isidore Moyenga, Martial Ouedraogo, Alberto Roggi, Nuccia Saleri, Elisa Tagliani, Enrico Tortoli

**Affiliations:** Istituto di Ricovero e Cura a Carattere Scientifico San Raffaele, Milan, Italy (E. Borroni, D.M. Cirillo, E. Tagliani, E. Tortoli);; University of Ouagadougou Yalgado National Hospital, Ouagadougou, Burkina Faso (G. Badoum, M. Quedraogo);; University of Brescia, Brescia, Italy (A. Matteelli, A. Roggii, N. Saleri) Ministry of Health, Ouagadougou (I. Moyenga

**Keywords:** Mycobacterium sherrisii, NTM pulmonary disease, MDR-TB, multidrug-resistant tuberculosis, Burkina Faso, nontuberculous mycobacteria, tuberculosis and other mycobacteria

**To the Editor**: Pulmonary disease caused by nontuberculous mycobacteria (NTM) is increasing worldwide. The most commonly recognized species, with minor geographic differences, are *Mycobacterium avium* complex (MAC), *M. kansasii*, *M. abscessus,* and *M. xenopi* (*1*). Little is known about the role of NTM in pulmonary disease in countries with a high prevalence of tuberculosis (TB). In such settings, smear microscopy (to identify acid-fast bacilli) is the primary, and often the only, diagnostic tool used to diagnose presumptive TB. Therefore, pulmonary disease caused by NTM is frequently misdiagnosed as TB and, because of the patient’s lack of response to standard anti-TB treatment, as multidrug-resistant TB (*2*).

In 2012, to detect possible drug resistance, we selected patients with pulmonary TB at the pulmonology division of Ouagadougou University Hospital (Ouagadougou, Burkina Faso), whose cases were classified as failing category II treatment (for patients with history of previous TB treatment). Each patient provided 1 sputum sample for culture and first- and second-line drug susceptibility testing (DST). Culture and DST were performed at the Supranational Laboratory of Milan (Milan, Italy), which provides technical assistance to the National Reference Laboratory in Burkina Faso. Of 314 samples NTM grew in culture for 36 (11%). Most NTM were identified as MAC (20 isolates). In culture of samples from 4 of the remaining patients, *M. sherrisii* grew. We describe the epidemiologic and clinical characteristics of these 4 patients. 

Three patients were male. All were born and lived in Burkina Faso and were HIV-negative; their ages ranged from 33 to 57 years. All had a history of having received 2 courses (categories I and II) of treatment for pulmonary TB. All were symptomatic, and their sputum samples were highly positive for acid-fast bacilli (1–10 cells/field; Ziehl-Neelsen stain). One patient did not return for further evaluation after this early assessment; the 3 others underwent a chest radiograph that showed, for each, pulmonary lesions compatible with TB (Table). The clinical specimens investigated in Milan were negative for *M. tuberculosis* complex by specific PCR (GenoType MTBDR*plus*, Hain Lifesciences, Nehren, Germany) and grew NTM in culture. The strains were identified as *M. simiae* with GenoType Mycobacterium CM/AS line probe assay (Hain Lifesciences), but because of the known cross-reactivity of the *M. simiae*–specific probe in this kit (*3*), the 16S rRNA gene was sequenced. All strains showed 100% identity to *M. sherrisii* strain NLA000800640 (GenBank accession no. EU883389), a strain previously isolated from a patient in Tanzania (*4*). 

**Table:** Clinical features of and microbiological findings from 4 patients with *Mycobacterium sherrisii* infection, Burkina Faso, 2012*

Patient no.	Age, y/sex	HIV status	Smear results	TB treatments	NTM treatment begun	Radiology	Outcome
1	40/F	Negative	3+	2011, 2012	October 2012	Cavitation in the right upper lobe. Bilateral apical bronchopneumonia with small pleural effusion in the right lung and basal emphysema.	Lost to follow-up
2	33/M	Negative	2+	2011, 2012	October 2012	Bilateral apical bronchopneumonia with consolidation in the middle lobe.	Died
3	57/M	Negative	2+	2012	NA	NA	Lost to follow-up
4	36/M	Negative	3+	2011, 2012	December 2012	Massive bilateral pneumonia	Died
*TB, tuberculosis; NTM, nontuberculous mycobacteria; NA, not applicable.							

On the basis of these findings, a treatment regimen that included clarithromycin was begun for the 3 patients, in addition to the anti-TB regimen with isoniazid, rifampin, and ethambutol. One patient was lost to follow-up during the first 2 months of treatment and the 2 others died. Further information was available for only 1 of those who died: he died of heart failure after 9 months of treatment. The pulmonary disease may well have been the cause; no autopsy was performed.

The presence of clear signs and symptoms compatible with pulmonary TB and the contemporary exclusion of *M. tuberculosis*, supported by the unresponsiveness to specific treatments and by the negative PCR results of strongly smear-positive sputum samples clearly fulfill the clinical criteria of the America Thoracic Society for NTM pulmonary disease (*5*). Meeting the objective of a second isolation, as required by microbiological criteria, was not possible because a second sputum sample was unavailable.

*M. sherrisii* is a relatively new species (*6*), closely related to *M. simiae*. Although most of the rare *M. sherrisii* infections reported since 2004 (Technical Appendix Table 1) were diagnosed in Europe or the United States, about half of the strains were isolated from patients in Africa. Because *M. sherrisii *infection probably is further underestimated by being misidentified as *M. simiae* infection by the commercially available line probe assays, the hypothesis that *M. sherrisii* infection is not so infrequent in the African setting seems therefore reasonable. In addition, the strategy recommended by World Health Organization and based on use of immunochromatographic tests (*7*), does not enable NTM identification. A leitmotiv of most *M. sherrisii* infections reported to date is HIV co-infection, which leads to dissemination of the mycobacterial disease. 

This report, although it adds to the record of patients in Africa, does not support the association with HIV infection. Our findings are consistent with the view that the pathogenic potential of *M. sherrisii* is comparable to that of other well-known NTM species (e.g., MAC) responsible for disease both in HIV-positive and HIV-negative patients. The retrospective determination of the MICs of antimicrobial agents potentially active against slowly growing mycobacteria (Technical Appendix Table 2) confirmed, for the 4 strains of *M. sherrisii*, the well-known multidrug resistance of the species (*8*). The therapeutic failure was thus not surprising because clarithromycin was the only drug among those administered during the treatment that had been shown to be active in vitro. This report provides evidence that conducting appropriate microbiological investigations is essential before initiating a treatment with second-line TB drugs (*9*).

Technical Appendix Tables for *Mycobacterium sherrisii *infections reported to date, and results of drug susceptibility testing for the 4 identified strains of *Mycobacterium sherrisii*, Burkina Faso, 2012.

## References

[1] Hoefsloot W, van Ingen J, Andrejak C, Angeby K, Bauriaud R, Bemer P, The geographic diversity of nontuberculous mycobacteria isolated from pulmonary samples: an NTM-NET collaborative study. Eur Respir J. 2013;42:1604–13. 10.1183/09031936.00149212 23598956

[2] Badoum G, Saleri N, Dembele MS, Ouedraogo M, Pinsi G, Boncoungou K, Failing a re-treatment regimen does not predict MDR/XDR tuberculosis: is “blind” treatment dangerous? Eur Respir J. 2011;37:1283–5. 10.1183/09031936.00144710 21532018

[3] Tortoli E, Pecorari M, Fabio G, Messinò M, Fabio A. Commercial DNA-probes for mycobacteria incorrectly identify a number of less frequently encountered species. J Clin Microbiol. 2010;48:307–10. 10.1128/JCM.01536-09 19906898 PMC2812265

[4] Crump JA, van Ingen J, Morrissey AB, Boeree MJ, Mavura DR, Swai B, Invasive disease caused by nontuberculous mycobacteria, Tanzania. Emerg Infect Dis. 2009;15:53–5. 10.3201/eid1501.081093 19116050 PMC2660713

[5] Griffith DE, Aksamit T, Brown-Elliott BA, Catanzaro A, Daley C, Gordin F, An official ATS/IDSA statement: diagnosis, treatment, and prevention of nontuberculous mycobacterial disease. Am J Respir Crit Care Med. 2007;175:367–416. 10.1164/rccm.200604-571ST 17277290

[6] Selvarangan R, Wu WK, Nguyen TT, Carlson LDC, Wallis CK, Stiglich SK, Characterization of a novel group of mycobacteria and proposal of *Mycobacterium sherrisii* sp. nov. J Clin Microbiol. 2004;42:52–9. 10.1128/JCM.42.1.52-59.2004 14715731 PMC321678

[7] World Health Organization. Use of liquid TB culture and drug susceptibility testing (DST) in low and medium income settings. Geneva: the Organization; 2007.

[8] van Ingen J, Tortoli E, Selvarangan R, Coyle MB, Crump JA, Morrissey AB, *Mycobacterium sherrisii* sp. nov.; a slow-growing non-chromogenic species. Int J Syst Evol Microbiol. 2011;61:1293–8. 10.1099/ijs.0.024752-0 20639227 PMC3167899

[9] Miotto P, Saleri N, Dembele M, Ouedraogo M, Badoum G, Pinsi G, Molecular detection of rifampin and isoniazid resistance to guide chronic TB patient management in Burkina Faso. BMC Infect Dis. 2009;9:142. 10.1186/1471-2334-9-142 19715569 PMC2739213

