# Supplementary material for: Mycobacterium sherrisii Pulmonary Disease, Burkina Faso
Source: Emerg Infect Dis. 2015 Nov;21(11):2093–4. doi: 10.3201/eid2111.141809 (PMC4622231; doi:10.3201/eid2111.141809)
Supplement: Technical Appendix — Tables for Mycobacterium sherrisii infections reported to date, and results of drug susceptibility testing for the 4 identified strains of Mycobacterium sherrisii, Burkina Faso, 2012. [file 14-1809-Techapp-s1.pdf]

DOI: http://dx.doi.org/10.3201/eid2111.141809

Mycobacterium sherrisii Pulmonary Disease, Burkina Faso

Technical Appendix

Technical Appendix Table 1. *Mycobacterium sherrisii* infections reported to date

Reference	Patient origin*	HIV status*	Disease*
Gamperli A, et al. J.Clin.Microbiol. 2005; 43:4283–5	Africa	Positive	Pulmonary
Tortoli E, et al. AIDS 2007; 21:1496–7	Italy (2)	Negative (2)	Pulmonary (2)
Loulergue P, et al. AIDS 2007;21:893–4	France, lived in Africa for 30 y	Positive	Pulmonary
Tortoli E, et al. Diagn.Micobiol.Infect.Dis. 2007; 57:221–3	Eritrea	Positive	Disseminated
Crump JA, et al. Emerg.Infect.Dis. 2009; 15:53–5	Tanzania (2)	Positive (2)	Disseminated (2)
Barrera L. et al. Medicina. 2010; 70:343–6	Argentina (6)	Negative (3), positive (3)	Pulmonary (3), pulmonary (2), disseminated (1)
Ho J, et al. Int.J.STD AIDS 2012; 23:369–0	Singapore	Positive	Disseminated
Tajan J, et al. Am.J.Trop.Med.Hyg. 3013; 88:914–7	Ghana	Positive	Disseminated + pulmonary
Lai C, et al. Int.J.Infect.Dis. 2014; 5:119–21	Africa	Positive	Pulmonary
This report	Burkina Faso (4)	Negative (4)	Pulmonary (4)

*Number of patients, if >1, is in parentheses.

Technical Appendix Table 2. Results of drug susceptibility testing for the 4 identified strains of *Mycobacterium sherrisii*, Burkina Faso, 2012*

	MICs by patient specimen, µg/mL				
Drug	Patient 1	Patient 2	Patient 3	Patient 4	Interpretation†
Amikacin	16	16	32	16	I
Ciprofloxacin	>16	>16	>16	>16	R
Clarithromycin	8	8	4	4	S
Doxycycline	>16	16	≥16	≥16	R
Ethambutol	16	16	16	16	R
Isoniazid	>8	>8	>8	>8	R
Linezolid	16	32	32	32	I-R
Moxifloxacin	8	4	8	8	R
Rifabutin	0.50	0.25	1	0.50	S
Rifampin	>8	8	>8	>8	R
Streptomycin	64	>64	64	64	R
Sulfamethoxazole	>152	>152	>152	>152	R

*I, intermediate; R, resistant; S, susceptible.
†In absence of criteria specific for *M. sherrisii* and related species, the cutoffs suggested by Clinical and Laboratory Standards Institute for other mycobacteria were used to indicate the MICs (*10*).

References

1. Gamperli A, Bosshard PP, Sigrist T, Brandli O, Wildermuth S, Weber R, et al. Pulmonary *Mycobacterium sherrisii* infection in a human immunodeficiency virus type 1-infected patient. J Clin Microbiol. 2005;43:4283–5. PubMed http://dx.doi.org/10.1128/JCM.43.8.4283-4285.2005

2. Tortoli E, Galli L, Andebirhan T, Baruzzo S, Chiappini E, de Martino M, et al. The first case of *Mycobacterium sherrisii* disseminated infection in a child with AIDS. AIDS. 2007;21:1496–8. PubMed http://dx.doi.org/10.1097/QAD.0b013e328235a53c

3. Loulergue P, Lamontagne F, Vincent V, Rossier A, Pialoux G. *Mycobacterium sherrisii*: a new opportunistic agent in HIV infection? AIDS. 2007;21:893–4. PubMed http://dx.doi.org/10.1097/QAD.0b013e3280f7750f

4. Tortoli E, Mariottini A, Mazzarelli G. *Mycobacterium sherrisii* isolation from a patient with pulmonary disease. Diagn Microbiol Infect Dis. 2007;57:221–3. PubMed http://dx.doi.org/10.1016/j.diagmicrobio.2006.06.021

5. Crump JA, van Ingen J, Morrissey AB, Boeree MJ, Mavura DR, Swai B, et al. Invasive disease caused by nontuberculous mycobacteria, Tanzania. Emerg Infect Dis. 2009;15:53–5. PubMed http://dx.doi.org/10.3201/eid1501.081093

6. Barrera L, Palmero D, Paul R, Lopez B. Enfermedad por Mycobacterium simiae y "*Mycobacterium sherrisii*" en la Argentina. Medicina (B Aires). 2010;70:343–6. PubMed

7. Ho J, Balm M, Huggan P, Chew N, Venkatachalam I, Archuleta S. Immune reconstitution inflammatory syndrome associated with disseminated *Mycobacterium sherrisii* infection. Int J STD AIDS. 2012;23:369–70. PubMed http://dx.doi.org/10.1258/ijsa.2011.011311

8. Tajan J, Espasa M, Sala M, Navarro M, Font B, Gonzalez-Martin J, et al. Disseminated infection by *Mycobacterium sherrisii* and *Histoplasma capsulatum* in an African HIV-infected patient. Am J Trop Med Hyg. 2013;88:914–7. PubMed http://dx.doi.org/10.4269/ajtmh.12-0572

9. Lai C, Lam W, Lee MP. A case of *Mycobacterium sherrisii* pneumonia diagnosed by PCR/ESI-MS method. Int J Infect Dis. 2014;25:119–21. PubMed http://dx.doi.org/10.1016/j.ijid.2014.03.1391

10. Clinical Laboratory Standards Institute. Susceptibility testing of mycobacteria, nocardiae and other aerobic actinomycetes; approved standard - Second Edition. M24-A2. Wayne (PA): CLSI; 2011.
